# Cardiomyocyte overexpression of microRNA-210 mitigates apoptotic cell death induced by doxorubicin

**DOI:** 10.1186/s40959-025-00429-z

**Published:** 2025-12-30

**Authors:** Johan Guthormsen, Mikal Solstad Øiaas, Mido Magdi Allam, Gurdeep Marwarha, Morten Andre Høydal

**Affiliations:** Department of Circulation and Medical Imaging, Norwegian University of Science and Technology, Faculty of Medicine and Health Sciences, Dept. of Circulation and Medical Imaging, PO Box 8905, Trondheim, NO-7491 Norway

**Keywords:** Doxorubicin, Apoptosis, Cardiotoxicity, MicroRNA

## Abstract

**Background:**

Advancements in cancer diagnostics and treatments have significantly increased patient survival rates. However, these advancements are often accompanied by treatment-induced health issues. Among these, chemotherapy-induced cardiac damage is particularly concerning, with doxorubicin being notably cardiotoxic. Despite extensive research, effective strategies to protect the heart from doxorubicin-induced cardiotoxicity remain elusive. This study aims to investigate whether miR-210, which has previously shown cardioprotective properties against ischemic heart disease, can offer protection against doxorubicin-induced cardiotoxicity.

**Methods:**

miR-210 was upregulated or downregulated in AC-16 cardiomyocytes using transient expression or decoy/inhibitory transfection vectors before being subjected to 5 µM doxorubicin treatment for 24 h. Cell death was determined using a lactate dehydrogenase release assay. Apoptotic cell death was determined by a caspase-3 activity assay, and the Akt-GSK-3β signaling pathway was explored using a sandwich enzyme-linked immunosorbent assay (ELISA) approach.

**Results:**

Overexpression of miR-210 in AC-16 cardiomyocytes exposed to 24 h of doxorubicin treatment caused a significant reduction in cell death and a significant reduction in apoptotic cell death, measured by caspase-3 activity. Downregulation of miR-210 in AC-16 cardiomyocytes exposed to the same conditions resulted in a significant increase in apoptotic cell death. An increase in phosphorylation of GSK-3β at the inhibitory p-Ser9 site and the Akt activating site p-Ser473 was observed in the miR-210 overexpression group, while a decrease in p-Ser473 Akt but no difference in p-Ser9 GSK-3β was observed in the miR-210-downregulated group.

**Conclusion:**

miR-210 exerts cardioprotective effects in AC-16 cardiomyocytes exposed to 24 h of doxorubicin treatment by reducing cell death and inhibiting caspase-3 dependent apoptosis through modulation of the Akt-GSK-3β signaling pathway. This study suggests a novel role for miR-210 in mitigating DOX-induced cardiomyocyte death, potentially laying the foundation for new treatment strategies.

## Introduction

Doxorubicin (DOX) is an anthracycline chemotherapeutic agent extensively used as a first-line treatment for various hematologic malignancies and solid tumors. It has demonstrated efficacy against numerous adult and pediatric cancers, even at lower doses. Furthermore, DOX is among the most potent anticancer drugs in clinical use, with resistance observed in only a few cancer types [1–3]. DOX is a member of the anthracycline class of cytotoxic antibiotics, which act by intercalating into DNA, inhibiting topoisomerase enzymes, disrupting mitochondrial function, and increasing free-radical production and oxidative damage [4]. Due to the observed side effects and mechanisms of action of DOX, there is now a broader focus in cancer treatment. This focus not only aims for cancer remission but also considers the quality of life for long-term survivors. A major drawback of DOX treatment is its inherent dose-dependent cardiotoxicity, which leads to clinical decompensation in 2–4% of patients, sub-clinical structural changes in 9–11%, and arrhythmias in over 12% of treated patients [5]. The cardiotoxic effects of DOX are among the most serious adverse events, significantly limiting its clinical utility. DOX can lead to progressive secondary cardiomyopathy, a disease of the heart muscle caused by extravascular factors. This condition may eventually progress to congestive heart failure, which carries a poor prognosis [6, 7]. However, patients without additional risk factors typically tolerate cumulative DOX doses of up to 300 mg/m^2^ moderately well, with a reported heart failure rate of less than 2% [8]. Exceeding this dosage threshold, however, markedly increases the rates of cardiotoxicity, which rise exponentially [9]. Moreover, numerous risk factors have been associated with increased sensitivity to developing DOX-induced cardiotoxicity; these include age, genetic predisposition, co-existing cardiovascular diseases, concurrent mediastinal radiation therapy, and combination with other chemotherapeutic agents [9]. Especially, elderly individuals (> 65 years of age) and children may be susceptible to developing clinical symptoms of cardiomyopathy at lower cumulative doses. In patients with childhood acute lymphoblastic leukemia treated with DOX, survivors showed persistent cardiac abnormalities such as reduced left ventricular contractility and abnormal left ventricular structure, which progressively worsened over time [10]. This occurred even at a cumulative dose of less than 300 mg/m^2^ [10]. Due to these deleterious effects, it is advised that the lifetime cumulative dose of DOX not exceed 400–450 mg/m^2^ [11].

Mechanistically, DOX-induced cardiotoxicity is an intricate process involving free radical-induced oxidative stress, dysregulation of calcium handling, adrenergic dysfunction, and selective inhibition of cardiomyocyte-specific gene expression [12]. Given its especially high mitochondrial density/volume compared to other organs, the heart is particularly sensitive to the oxidative damage caused by DOX, as mitochondria are both major sources and targets of reactive oxygen species (ROS) [13]. Through a process termed redox cycling, which involves the reduction and re-oxidization of DOX mediated by cellular oxidoreductase enzymes, ROS such as the superoxide anion radical (O_2_^−^) and hydrogen peroxide (H_2_O_2_) are generated [11, 14]. These molecules can further be converted to the highly reactive and toxic hydroxyl radicals ($\bullet$ OH), which, upon oxidation of cellular components, result in severe cellular damage [11].

Moreover, DOX also causes oxidative stress through other mechanisms, such as iron complexation and uncoupling of the electron transport chain in the mitochondria, and by disrupting antioxidant defense systems [15]. Additionally, in comparison with other tissues, the heart has relatively low levels of antioxidant-producing enzymes, such as peroxidase, superoxide dismutase, and catalase, making it even more vulnerable to DOX-induced oxidative stress [16, 17]. In cardiomyocytes, DOX targets the enzyme topoisomerase-IIβ, and the resulting topoisomerase-IIβ-DOX-DNA complex can cause DNA double-strand breaks [18]. Most of these cellular events trigger signaling pathways that culminate in cardiomyocyte death, which is a major contributor to DOX-induced cardiomyopathy [12]. The most studied cell death pathway in DOX-induced cardiomyocyte death is apoptosis [19]. In addition to apoptosis, DOX-induced cardiotoxicity also involves other regulated and unregulated cell death cell-death mechanisms [19], including autophagy [20–22], ferroptosis, [19, 23–25] and necroptosis [19, 26, 27]. Despite extensive research, current treatment options remain inadequate, underscoring the need for novel cardioprotective strategies.

In recent years, microRNAs (miRs) have emerged as promising modulators of DOX-induced cardiotoxicity [28]. As miRs generally have multiple target mRNAs, their potential to influence processes initiated by DOX is substantial. Moreover, several miRs have been shown to affect DOX-induced cardiotoxicity by targeting pathways involved in oxidative stress, apoptosis, mitochondrial function, and DNA methylation [28, 29]. This regulatory mechanism is essential for maintaining cellular homeostasis and responding to environmental changes. Consequently, miRs have emerged as potential biomarkers for disease diagnosis and prognosis, as well as novel therapeutic targets [30].

Among these, miR-210 stands out due to its consistent upregulation in hypoxic and ischemic conditions and its well-documented cardioprotective effects [31]. In a hypoxic environment, miR-210 initiates adaptive cellular responses and is implicated in processes such as angiogenesis, mitochondrial metabolism, cell cycle regulation, and apoptosis [32]. Through inhibition of apoptosis and promoting angiogenesis, miR-210 is believed to protect the cardiovascular system from potentially irreversible damage upon insufficient oxygen supply [33]. During hypoxia, miR-210 has been shown to inhibit apoptosis in human pulmonary artery smooth muscle cells through the downregulation of E2F transcription factor 3 (E2F3) [34]. Additionally, miR-210 has been demonstrated to inhibit apoptosis under hypoxic conditions in other cell types, such as in neural progenitor cells [35]. In AC 16 cardiomyocytes, miR-210 has been shown to attenuate the hypoxia-driven intrinsic apoptosis pathway [36].

Importantly, inhibition of glycogen synthase kinase-3β (GSK-3β) is a key mechanism by which miR-210 mitigates intrinsic apoptosis during hypoxia [37]. GSK-3β, is negatively regulated by Ser9 phosphorylation and positively by Tyr216 phosphorylation [38–41]. In DOX-treated cardiomyocytes, reduced pSer9 GSK-3β levels indicate increased GSK-3β activity [42]. Akt, a primary kinase in this pathway, phosphorylates GSK-3β at Ser9, and emerging evidence indicates that miR-210 regulates upstream components of Akt signaling [43–47].

However, no studies have yet determined whether miR-210 influences GSK-3β activity or modulates Akt signaling, a central regulator of cell survival, in the context of DOX-induced cardiotoxicity. Given this gap in knowledge, and the established role of miR-210 in cardioprotection, we hypothesized that miR-210 may attenuate DOX-induced cardiomyocyte apoptosis by enhancing Akt activation and inhibiting GSK-3β activity through the Akt–GSK-3β signaling axis.

Therefore, the present study aimed to investigate the potential protective effects of miR-210 in DOX-treated AC-16 cardiomyocytes, focusing specifically on its impact on Akt phosphorylation at Ser473, a key activation site, and subsequent inhibition of GSK-3β via Ser9 phosphorylation. This approach may provide novel insights into the molecular mechanisms underlying miR-210-mediated cardioprotection and identify new therapeutic targets for mitigating DOX-induced cardiac damage.

## Methods

### Cells and subculturing

Human AC-16 cardiomyocytes (EMD Millipore/Merck Millipore/Merk Life Sciences, Catalogue # SCC109, Darmstadt, Germany, RRID:CVCL_4U18) were cultured in Dulbecco’s Modified Eagle’s Medium Nutrient Mixture F-12 (DEM/F12), containing 12.5% fetal bovine serum (FBS) and 1% Antibiotic Antimycotic mix. AC-16 cardiomyocytes plated and subcultured in 100-mm cell culture plates incubated at 37℃ with 5% CO_2_. A total of 20 cell culture plates with a confluency of $\sim$ 80% were prepared, and 16 plates were chosen at random to be included in the respective experiments. The cells had a passage between 6 and 9 before the transfection step.

### Transfection procedure

When the sub cultured cells were $\sim$ 80% confluent, they were transiently transfected with the miR-210 expression vector (overexpression (OE) vector)(pEZX-MR04) (GeneCopoeia™, Rockville, MD, USA, Catalogue # HmiR0167-MR04) or the miR-210-3p *decoy/inhibitor* vector (knockdown (KD) vector)(pEZX-AM01-miR-210) (GeneCopoeia™, Rockville, MD, USA, Catalogue # HmiR-AN0317-AM01). For the control groups, an empty vector (EV) (pEZX-MR04-Scrambled)(GeneCopoeia™, Rockville, MD, USA, Catalogue # CmiR0001-MR04) was used in the mir-210 OE (miR-210 OE) experiment, while another empty vector (pEZX-AM01-Scrambled) (GeneCopoeia™, Rockville, MD, USA, Catalogue #) CmiR-AN0001-AM01) was used in the KD experiment.

A transfection mix containing 3 µg plasmid (vector), 15 µL Polyfect® transfection reagent (Qiagen Norge, Oslo, Norway, Catalogue # 301,107) and 150 µL plain media (DMEM without FBS and Antibiotic Antimycotic Solution) was prepared to transfect one 100-mm cell culture plate (approximately 6,000,000 cells). Therefore, a transfection mix enough for 10 plates was prepared for each group; that is, 30 µg plasmid, 150 µL transfection reagent, and 1.5 mL plain media. Firstly, the plasmid and the transfection reagent were mixed in a 1.5-mL tube. Thereafter, plain media was added to the mix, and the tube was incubated for 30 min at 37 ℃. While the transfection mix was incubating, the cells were centrifuged at 500 × g and pelleted. A total of 16 cell culture plates (100 mm) were segregated into 4 experimental groups, in which 2 groups (*n* = 4 per group) received a transfection mix containing the miR-210 OE vector and 2 groups (*n* = 4 per group) received a transfection mix containing EV. Each group received a total of 334 µL of the designated transfection mix (167 µL × 2). Subsequently, the cells were replated and incubated overnight in the incubator at 37 ℃ with 5% CO_2_. The equivalent strategy was employed in the miR-210 KD experiment.

### Doxorubicin treatment

In this study, human AC-16 cardiomyocytes were treated with DOX concentration of a terminal concentration of 5 µM Doxorubicin hydrochloride (DOX) (Sigma Aldrich, Darmstadt, Germany, Catalogue # 44,583-1MG) for 24 h. The 5 µM terminal concentration of DOX and the duration of the DOX treatment were chosen on the basis of previously established protocol that displayed robust response on LDH release to DOX treatment [48]. Firstly, a 1 mM DOX stock solution was prepared with sterile 18MΩ MilliQ H_2_O as a solvent. Then, 250 µL 1 mM DOX stock solution was added to 49.75 mL culture medium, creating a terminal 5 µM DOX solution in the cell culture medium. For the control groups, 250 µL sterile 18MΩ MilliQ H_2_O was added to 49.75 mL culture medium, creating a “vehicle” solution. Treated cell culture plates were incubated for 24 h in the incubator at 37 ℃ with 5% CO_2_. The equivalent strategy was employed for both miR-210 OE and the miR-210 KD experiments.

### Harvesting the cells and preparation of cell lysates

After 24-h of DOX treatment, cells were harvested and the cell lysates prepared, as follows. The conditioned medium was aspirated and collected in 50-mL tubes used for the Lactate dehydrogenase (LDH)-release assay. Subsequently, 1 mL of non-denaturing lysis buffer (20 mM Tris, 137 mM Nacl, 2 mM EDTA, 1% Nonidet P-40, 10% glycerol; pH 7.4) was added to each plate, and the cells were collected in 1.5-mL tubes. Next, the lysed cells were centrifuged at 12,000 × g 4 ℃ for 10 min. Following centrifugation, cell lysate (supernatant) was separated from cell debris (pellet), and the cell lysate was collected in new 1.5-mL tubes, while the cell debris was discarded. Total protein content of cellular lysates was quantitatively determined by the Bradford method [49] using the Bradford dye, *Coomassie brilliant blue G-250*, procured as a commercially formulated kit (Pierce™ Detergent Compatible Bradford Assay Kit, Thermo Fisher Scientific, Oslo, Norway, Catalogue # 23,246).

### Sandwich enzyme-linked immunosorbent assays (ELISA)

Downstream tandem sandwich ELISA immunoassays were employed as a quantitative detection method in the LDH release assay, miR-210 hybridization assay, as well as a direct method to determine the relative expression levels of Akt, p-Ser^473^ Akt, GSK3β and p-Ser9 GSK3β. The sandwich ELISA immunoassays were performed as previously described [36]. Briefly, 10–30 ng of the respective *capture* antibodies (Table 1) were immobilized in each well of the respective 96-well microplates. The respective cell lysates (equivalent to 10 μg of total protein content, determined by the Bradford Assay method [49]) or the conditioned media (50 µL) were incubated with the respective immobilized *capture* antibodies overnight at 4 °C. Subsequently, the respective 96-well microplate wells were washed 3 × (15 min each) with TBS-T (Tris-buffered saline with Tween) buffer (150 mM NaCl, 50 mM Tris, 0.1 v/v Tween-20, pH 7.4) and incubated with the respective *detection* antibodies (Table 1) overnight at 4 °C. The 96-well microplate wells were washed 3 × (15 min each) with TBS-T followed by immunodetection with the AP (alkaline phosphatase)-conjugated secondary antibodies (Table 1), using the AP-substrate PNPP (p-nitrophenyl phosphate, disodium salt) (Thermo Fisher Scientific, Oslo, Norway, Catalogue # 37,621) as the chromophore for the colorimetric read-out (λ_405_). The antibody signal specificity was established by performing peptide blocking assays. The antibody blocking peptides corresponding to the specific epitopes for the respective detection antibodies used are enumerated in (Table 1). The respective absorbances from the peptide blocking assays were used for the experimental blank correction. Data are expressed as experimental blank-corrected O.D_405_ (optical density measured at λ_405_ nm) values from three technical replicates for each of the four biological replicates belonging to each experimental group (*n* = 4).

**Table 1:** List of antibodies and antibody-blocking peptides utilized in this study

Antibody	Use	Amount	Host	Manufacturer	Catalogue #
Akt	ELISA capture	10 ng/well	Mouse	Cell Signalling Technology	2920
Akt	ELISA detection	10 ng/well	Rabbit	Cell Signalling Technology	4691
Akt	Western Blot	5 µg	Rabbit	Cell Signalling Technology	4691
p-Ser473 Akt	ELISA capture	10 ng/well	Mouse	Cell Signalling Technology	4051
p-Ser473 Akt	ELISA detection	10 ng/well	Rabbit	Cell Signalling Technology	9271
p-Ser^473^ Akt	Western Blot	5 µg	Rabbit	Cell Signalling Technology	9271
β-Actin	ELISA capture	20 ng/well	Mouse	Santa Cruz Biotechnology	sc-47778
β-Actin	ELISA detection	20 ng/well	Rabbit	Cell Signalling Technology	4970
β-Actin	Western Blot	3 µg	Mouse	Santa Cruz Biotechnology	sc-47778
β-Actin antibody blocking peptide	ELISA detection	N/A	N/A	Cell Signalling Technology	1025
Digoxigenin	ELISA capture	15 ng/well	Rabbit	R&D Systems	MAB10386
Goat Anti-Mouse IgG (H + L)–HRP Conjugate	Western Blot	1:5000*	Goat	Bio-Rad	1,706,516
Goat Anti-Rabbit IgG (H + L)–HRP Conjugate	Western Blot	1:5000*	Goat	Bio-Rad	1,706,515
Goat Anti-Rabbit IgG-AP Conjugate	ELISA	1:20,000*	Goat	Sigma Aldrich/Merck Life Science	A3687
GSK-3β	ELISA capture	10 ng/well	Mouse	Cell Signalling Technology	9832
GSK-3β	ELISA detection	10 ng/well	Rabbit	Cell Signalling Technology	9315
GSK-3β	Western blot	4 µg	Rabbit	Cell Signalling Technology	9315
p-Ser9 GSK-3β	ELISA capture	10 ng/well	Mouse	Cell Signalling Technology	14,630
p-Ser9 GSK-3β	ELISA detection	10 ng/well	Rabbit	Cell Signalling Technology	9322
p-Ser^9^ GSK-3β	Western blot	5 µg	Rabbit	Cell Signalling Technology	9322
LDH	ELISA capture	30 ng/well	Mouse	Santa Cruz Biotechnology	sc-133123
LDH-A	ELISA detection	30 ng/well	Rabbit	Novus Biologicals	NBP1-48,336
LDH-A antibody blocking peptide	ELISA detection	N/A	N/A	Novus Biologicals	NBP1-48336PEP
LDH-B	ELISA detection	30 ng/well	Rabbit	Novus Biologicals	NBP2-38,131
LDH-B antibody blocking peptide	ELISA detection	N/A	N/A	Novus Biologicals	NBP2-38131PEP

*N/A* Not available/not applicable

^*^Amount of secondary antibody cannot be determined as the commercial vendor does not provide the antibody concentration

### Enzyme-linked miR-210 hybridization immunoassay

The enzyme-linked oligonucleotide hybridization assay (ELOHA) [50–54] approach was adopted to quantitatively determine miR-210 expression levels, as previously described [36]. This microRNA immunoassay approach enables the quantitative determination of miR-210 in the same experimental lysates being subjected to the specific downstream assays. In brief, streptavidin-immobilized biotin-conjugated miR-210 locked nucleic acid (LNA) capture probe (Qiagen Norge, Oslo, Norway, Catalogue # 339,412 YCO0212944) was utilized to affinity-capture miR-210 by hybridization. The resultant double-stranded affinity-captured miR-210 was denaturated at 90 °C for 30 min and striped-off the streptavidin beads (10 mM Tris, pH 7.5). The eluted miR-210 was solid-state immobilized in the 96-well microplates and hybridized with digoxigenin-labeled miR-210 LNA detection probe (Qiagen Norge, Oslo, Norway, Catalogue # 339,412 YCO0212945). The amount of the miR-210-bound digoxigenin-labeled LNA detection probe was immunodetected with the AP (alkaline phosphatase)-conjugated digoxigenin antibody (Digoxigenin AP-conjugated Antibody, R&D Systems, Minneapolis, MN, USA, Catalogue # APM7520) using the AP-substrate PNPP as the chromophore for the colorimetric read-out (optical density [O.D] at λ_405_). Data are expressed as a fold-change ± standard deviation (S.D) of the O.D_405_ (λ_405_) values from three technical replicates for each of the four biological replicates belonging to each experimental group (*n* = 4).

### Lactate dehydrogenase (LDH)-release assay

The LDH-release assay was performed by adding 50 µL of the respective conditioned media and 150 µL PBS (phosphate-buffered saline – 137 mM Nacl, 2.7 mM Kcl, 10 mM Na_2_HPO_4_, 1.8 mM KH_2_PO_4_) buffer to each well of a 96-well microplate precoated with immobilized LDH *capture* antibody The immunoaffinity captured LDH was immunodetected using LDH-A and LDH-B *detection* antibodies (Table 1) following the protocol described above under the section *Sandwich enzyme-linked immunosorbent assays (ELISA).*

### Caspase-3 activity assay

The specific caspase-3 substrate Ac-DEVD-p-NA (n-Acetyl-Ac-Asp-Glu-Val-Asp-p-nitroanilide) is cleaved by caspase 3 and caspase 7 into a chromogenic reaction product, enabling colorimetric estimation of their activities [55]. Cell lysates (6 × 10^6^ cells per plate) were prepared in *non-denaturing* lysis buffer (20 mM Tris, 137 mM Nacl, 2 mM EDTA, 1% Nonidet P-40, 10% glycerol; pH 7.4). In the respective wells of a 96-well microplate (200 µL assay volume), an *input* corresponding to 200-µg equivalent of protein content was incubated with 40 nmoles of the caspase-3 substrate, Ac-DEVD-p-NA (Sigma Aldrich, Oslo, Norway, Catalogue # 235,400-5MG) for 4 h at 37 °C. The caspase-3 activity was determined by measuring the optical density (O.D) of the resultant chromophore (absorbance at λ_405_) produced. To exhibit assay specificity and serve as an experimental blank, assays were also performed in the presence of the caspase-3 inhibitor, Ac-DEVD-CHO (Sigma Aldrich/Merck Millipore/Merck Life Science, Darmstadt, Germany, Catalogue # 235,420), Data are expressed as a fold-change ± standard deviation (S.D) of the experimental blank-corrected O.D_405_ (λ_405_) values from three technical replicates for each of the four biological replicates belonging to each experimental group (*n* = 4).

### Western blotting

Treated AC-16 cells (6 × 10^6^ cells per plate) seeded in 100 mm plates, transfected, and subjected to the experimental interventions (as described earlier), were lysed in a *non-denaturing* lysis buffer (20 mM Tris, 137 mM Nacl, 2 mM EDTA, 1% Nonidet P-40, 10% glycerol; pH 7.4) supplemented with phosphatase inhibitors. Protein concentrations in the cell lysates was determined with the Bradford protein assay [49] using the “Pierce™ Detergent Compatible Bradford Assay Kit” (Thermo Fisher Scientific, Oslo, Norway, Catalogue # 23,246). Proteins (30 μg) were resolved on 10% SDS-PAGE gels [56, 57] followed by transfer onto a polyvinylidene difluoride (PVDF) membrane (Immun-Blot™ PVDF Membrane, Bio-Rad Norway AS, Oslo, Norway, Catalogue # 1,620,177). The Western blots were subjected to non-specific blocking with 5% BSA (Bovine Serum Albumin) for 1 h. Subsequently, the respective western blots were washed 5 × (10 min each) with TBS-T buffer and incubated with the respective primary antibodies (Table 1) overnight at 4 °C. The respective western blots were washed 3 × (10 min each) with TBS-T followed by incubation with HRP (Horseradish peroxidase)-conjugated secondary antibodies (Table 1) for 1 h. The respective western blots were washed 5 × (10 min each) with TBS-T buffer and developed with enhanced chemiluminescence substrate (SuperSignal™ West Pico PLUS Chemiluminescent Substrate, Thermo Fisher Scientific, Catalogue # 34,580). The western blots were imaged using a LICOR Odyssey Fc imaging system (LI-COR Biotechnology, Cambridge, UK). Quantitative densitometry was performed using Image J (ImageJ, United States National Institutes of Health, Bethesda, MD, USA, https://imagej.nih.gov/ij/) and the results analyzed as total integrated densitometric values.

### Bioinformatic predictions

To explore potential molecular interactions involving miR-210-3p, we initiated a bioinformatic workflow by identifying predicted human gene targets of hsa-miR-210-3p using the TargetScanHuman 8.0 database [58], which utilizes seed sequence complementarity and evolutionary conservation to predict microRNA–mRNA interactions. 42 transcripts with conserved sites, containing a total of 50 conserved sites and 4 poorly conserved sites targeted by mir-210-3p were found (*details in Supplementary table S1*). The resulting list of predicted targets was then analyzed in conjunction with Akt using the STRING v12.0 database [59] to explore potential protein–protein interaction networks and functional associations. Akt was included to specifically assess whether any of the predicted miR-210-3p targets are directly or indirectly linked to Akt signaling pathways.

STRING analysis was performed using default active interaction sources, including text mining, experimental data, curated databases, co-expression, gene neighborhood, gene fusion, and co-occurrence. A medium confidence threshold (interaction score ≥ 0.4) was applied to maximize detection of plausible interactions while maintaining biological relevance. This integrative approach enabled the identification of candidate genes that may serve as intermediaries between miR-210-3p and Akt, potentially contributing to the observed modulation of apoptotic signaling in DOX-treated cardiomyocytes.

### Statistical analysis

All values are presented as means ± standard deviation (S.D.). Data were analyzed using GraphPad Prism (version 10.1.2) statistical software. Multiple comparisons between the different treatment groups were performed using one-way ANOVA with Tukey’s Post Hoc test. For all tests, a p-value of less than 0.05 was defined as statistically significant. A total of 20 cell culture plates (AC-16 cardiomyocytes plated and subcultured in 100-mm cell culture plates) were prepared, and 16 plates were chosen at random to be included in the respective experiments. Each randomly selected plate served as one biological replicate that were split and subcultured in tree to serve as technical replicates for each experimental group. The choice of using four biological replicates was based on prior studies from our lab employing similar experimental designs and cell-based assays [36], with aim to provide sufficient statistical power to detect biologically meaningful differences in AC-16 cardiomyocytes and mitigate intra-assay variability, particularly when combined with three technical replicates per biological sample.

## Results

### miR-210 expression increased following DOX reatment

**Fig. 1:**
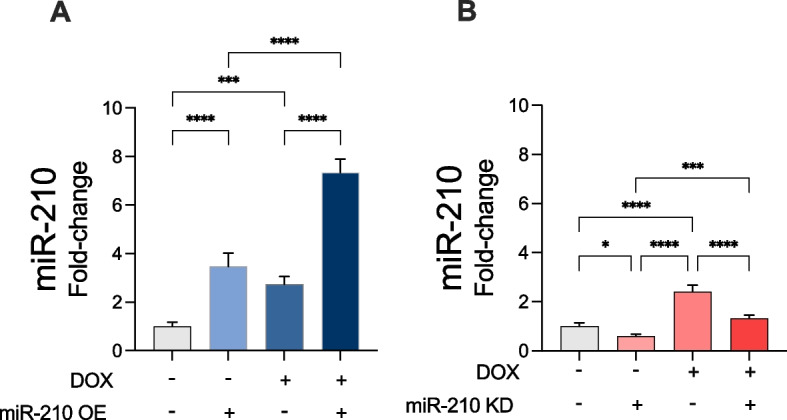
Validation of miR-210 expression levels in AC16 cells. miR-210 expression levels in the respective cell lysate inputs in all experimental groups were determined by the miR-210 hybridization immunoassay. **A** Effect of miR-210 overexpression (OE) and (**B**) effect of miR-210 knockdown (KD). Absorbance was measured at 405 nm. All data are expressed as mean ± standard deviation from three technical replicates for each of the four biological replicates (*n* = 4) belonging to each experimental group. * *p* < 0.05; *** *p* < 0.001; **** *p* < 0.0001. DOX: doxorubicin; OE: miR-210 overexpression; KD: miR-210 knockdown. Notation: “ + ” indicates treatment applied; “–” indicates absence of treatment. For DOX, “ + ” = DOX-treated, “–” = vehicle. For miR-210 OE, “ + ” = miR-210 OE, “–” = empty vector. For miR-210 KD, “ + ” = miR-210 knockdown, “–” = empty vector

To determine the expression levels of miR-210 following DOX treatment for verifying the efficiency of OE or KD of miR-210, we performed a miR-210 hybridization assay. In EV-transfected cells, we found a significant increase in miR-210 levels in cells treated with DOX compared to vehicle (*p* < 0.0001; Fig. 1A-B). Comparing vehicle-treated cells, transfection with miR-210 OE caused a significant increase of miR-210 compared to control (*p* < 0.0001; Fig. 1A). In DOX-treated cells, we found a significant increase in miR-210 levels in cells transfected with miR-210 OE compared to EV (*p* < 0.0001; Fig. 1A). We observed a significant decrease in miR-210 levels when transfecting with miR-210 KD in both vehicle-treated cells (*p* < 0.05; Fig. 1B) and DOX-treated cells (*p* < 0.0001) compared to transfecting with EV (Fig. 1B).

### miR-210 reduces cell death in DOX-treated AC-16 cardiomyocytes

**Fig. 2:**
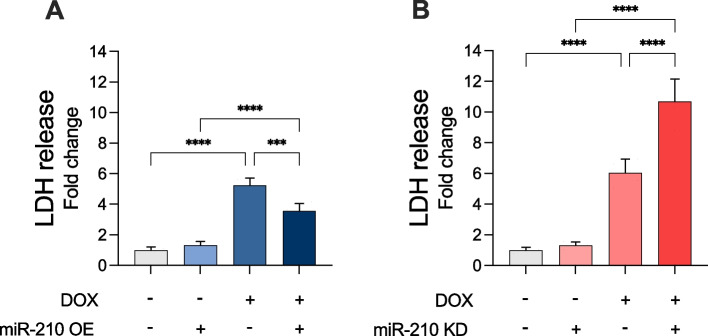
Overexpression and downregulation of miR-210 affect cell death in DOX-treated AC-16 cardiomyocytes. **A** miR-210 overexpression (OE) significantly reduced general cell death, while (**B**) miR-210 knockdown (KD) significantly increased general cell death, as measured by a lactate dehydrogenase (LDH) release assay. Absorbance was measured at 405 nm. All data are expressed as mean ± S.D. from three technical replicates for each of the four biological replicates (*n* = 4) belonging to each experimental group. *** *p* < 0.001; **** *p* < 0.0001. DOX: doxorubicin; LDH: lactate dehydrogenase; OE: miR-210 overexpression; KD: miR-210 knockdown; S.D.: standard deviation. Notation: “ + ” indicates treatment applied; “–” indicates absence of treatment. For DOX, “ + ” = DOX-treated, “–” = vehicle. For miR-210 OE, “ + ” = miR-210 OE, “–” = empty vector. For miR-210 KD, “ + ” = miR-210 knockdown, “–” = empty vector

To determine if OE or KD of miR-210 would affect cell death in AC-16 cardiomyocytes exposed to DOX treatment, an LDH-release assay was performed. Overexpression of miR-210 reduced the LDH release (*p* < 0.001; Fig. 2A), while downregulation of miR-210 augmented the LDH release (*p* < 0.0001; Fig. 2B) in the condition media after being treated with 5 µM DOX medium for 24 h. These results indicate that miR-210 reduces cell death in DOX-treated AC-16 cardiomyocytes.

### miR-210 inhibits apoptosis in DOX-treated AC-16 cardiomyocytes

**Fig. 3:**
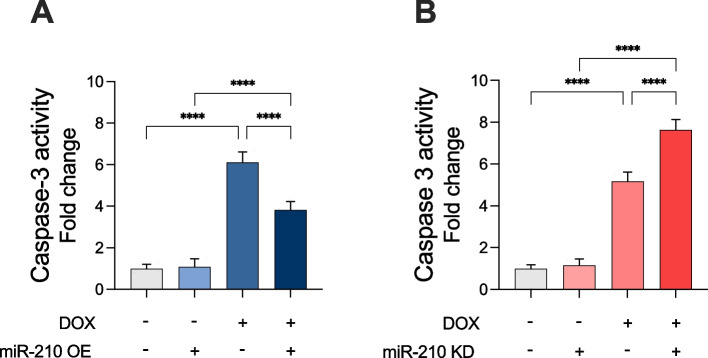
miR-210 reduces DOX-induced apoptotic cell death in AC-16 cardiomyocytes. **A** Overexpressing miR-210 (OE) significantly attenuated caspase-3 activity, while (**B**) knocking down miR-210 (KD) significantly increased caspase-3 activity. Absorbance was measured at 405 nm. Caspase-3 activity was corrected for total protein content in each sample measured by β- Actin. All data are expressed as mean ± S.D. from three technical replicates for each of the four biological replicates (*n* = 4) belonging to each experimental group. **** *p* < 0.0001. DOX: doxorubicin; OE: miR-210 overexpression; KD: miR-210 knockdown; S.D.: standard deviation. Notation: “ + ” indicates treatment applied; “–” indicates absence of treatment. For DOX, “ + ” = DOX-treated, “–” = vehicle. For miR-210 OE, “ + ” = miR-210 OE, “–” = empty vector. For miR-210 KD, “ + ” = miR-210 knockdown, “–” = empty vector

To further elucidate the findings that miR-210 reduces cell death, as indicated by LDH release, we measured caspase-3 activity. This was done to determine whether the observed reduction in cell death could be partially attributed to an effect on apoptotic pathways. Overexpression of miR-210 significantly reduced caspase-3 activity in AC-16 cardiomyocytes exposed to DOX (p < 0.0001), while no difference in caspase 3 activity was observed in the unstimulated vehicle group (Fig. 3A). We did also confirm that miR-210 KO increased caspase 3 activity significantly in the DOX-treated group (*p* < 0.0001). No difference in caspase 3 activity was observed in the vehicle group (Fig. 3B). These results indicate that miR-210 inhibits cell death in AC-16 cardiomyocytes through modulating apoptosis.

### miR-210 inhibits DOX-induced apoptotic cell death by affecting phosphorylation status of GSK-3β

**Fig. 4:**
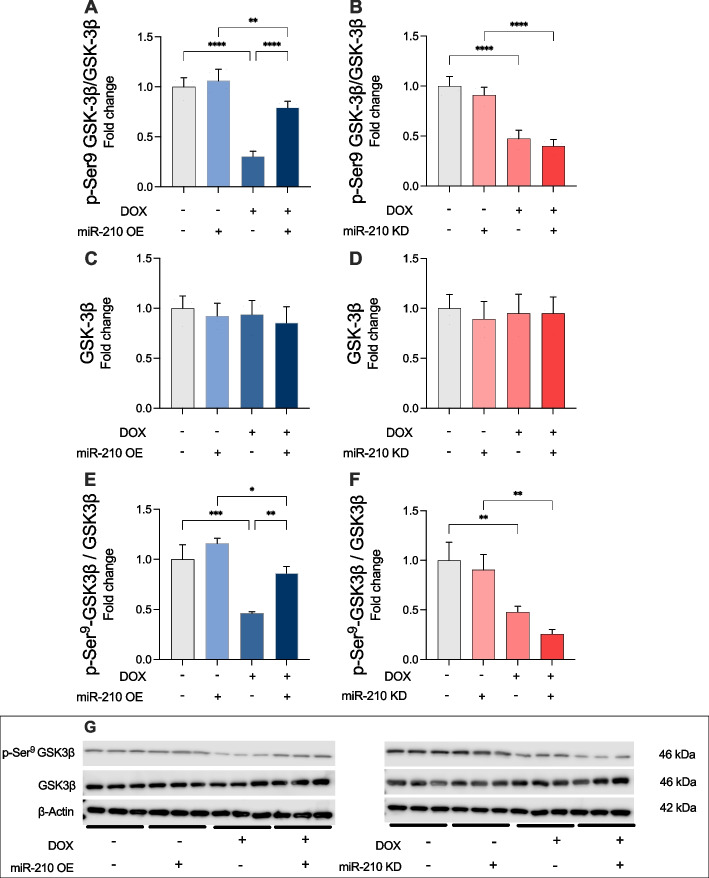
miR-210 overexpression increases phosphorylation at serine 9 residue (Ser9) of glycogen synthase kinase-3β (GSK-3β)in DOX-treated AC-16 cardiomyocytes. **A** Phosphorylation at Ser9 of GSK-3β is increased with miR-210 OE vector as compared to EV after 24-h DOX treatment. **B** No significant difference in phosphorylation at Ser9 of GSK-3β is observed with miR-210 KD vector as compared to EV after 24-h DOX treatment. No change in protein expression levels of GSK-3βwere observed in AC-16 cardiomyocytes transfected with an overexpression (OE) vector (**C**) nor a knockdown (KD) vector (**D**) of miR-210 across all treatment arms. Protein expression of phosphorylation at Ser9 of GSK-3β was further confirmed by western blot under the condition of (**E**) miR-210 OE and (**F**) mir-210 KD. Protein expression of GSK-3β was corrected for total protein content in each sample measured by β- Actin. Absorbance was measured at 405 nm. All data are expressed as mean ± S.D. All data for ELISA analyses (**A**-**D**) was run from three technical replicates for each of the four biological replicates (*n* = 4) belonging to each experimental group. Panel **G** display exemplary western blot. Samples for was performed on three random selected samples from each group presented in panel A-D to allow comparison on the same gel.** *p* < 0.01; **** *p* < 0.0001. DOX: doxorubicin; OE: miR-210 overexpression; KD: miR-210 knockdown; S.D.: standard deviation. Notation: “ + ” indicates treatment applied; “–” indicates absence of treatment. For DOX, “ + ” = DOX-treated, “–” = vehicle. For miR-210 OE, “ + ” = miR-210 OE, “–” = empty vector. For miR-210 KD, “ + ” = miR-210 knockdown, “–” = empty vector

Previous studies have demonstrated that miR-210 modulates hypoxia-induced apoptosis by affecting the phosphorylation status of GSK-3β at Ser9. To investigate whether a similar mechanism underlies the miR-210-mediated decrease in DOX-induced apoptosis, we quantified the levels of GSK-3β protein in lysates from DOX-treated AC-16 cardiomyocytes. These cells were transfected with either an OE vector or a KD vector. We found that transfection with a miR-210 OE vector caused a significant increase in p-Ser9 GSK-3β compared to the EV control group after DOX treatment (p < 0.0001; Fig. 4A). In contrast, no significant difference in relative p-Ser9 GSK-3β levels was observed when miR-210 was knocked down as compared to the EV control group after DOX treatment (Fig. 4B). Total protein expression levels of GSK-3β were not different between any groups (Fig. 4C and D). GSK-3β Ser9 phosphorylation status was also confirmed by western blot (Fig. 4E-G). These results indicate that miR-210 increases phosphorylation of GSK-3β at Ser9 with consequences of reduced GSK-3β activity in cells exposed to DOX.

### miR-210 affects phosphorylation status of Akt in DOX-treated AC-16 cardiomyocytes

**Fig. 5:**
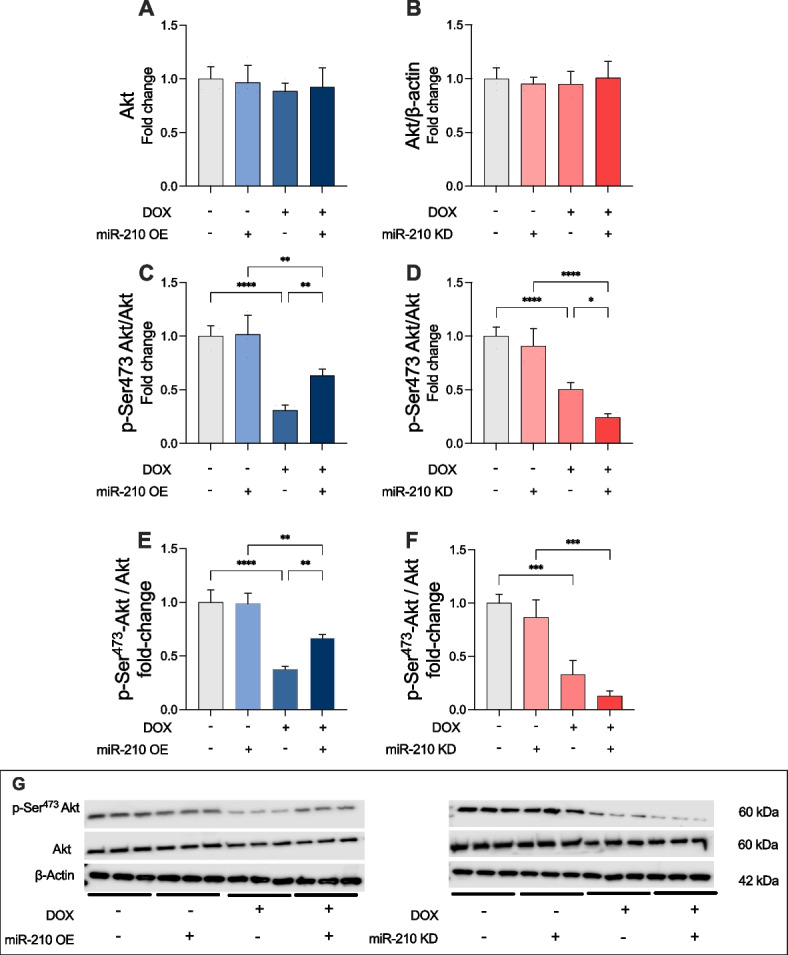
miR-210 affects phosphorylation of Akt at serine 473 (Ser473) in DOX-treated AC-16 cardiomyocytes. No change in protein expression levels of Akt were observed in AC-16 cardiomyocytes transfected with either (**A**) the overexpression (OE) vector of miR-210 or the knock down vector (KD) (**B**). **C** Phosphorylation at Ser473 of Akt is increased with miR-210 OE vector as compared to EV after 24-h DOX treatment. **D** A significantly lower degree of phosphorylation at Ser473 of Akt is observed with miR-210 KD vector as compared to empty vector (EV) after 24-h DOX treatment. All data for ELISA analyses was run from three technical replicates for each of the four biological replicates (*n* = 4) belonging to each experimental group. Expression levels of phosphorylation at Ser473 of Akt was further confirmed by western blot under the condition of (**E**) the overexpression miR-210 OE and (**F**) mir-210 KD. Absorbance was measured at 405 nm. Protein expression of Akt was corrected for total protein content in each sample measured by β- Actin that also served as control for expression of GSK-3β protein loading. Panel **G** display exemplary western blot. Samples for was performed on three random selected samples from each group presented in panel A-D to allow comparison on the same gel. * *p* < 0.05; ** *p* < 0.01; **** *p* < 0.0001. DOX: doxorubicin; OE: miR-210 overexpression; KD: miR-210 knockdown; S.D.: standard deviation. -/+ to each group for DOX denotes if the condition of the cells were with DOX (+) or with vehicle treatment (-). For miR-210 OE -/+ denotes if cells where treated by miR-210 OE (+) or denotes the treatment were EV (-). For miR-210 KD -/+ denotes if cells were treated by miR-210 KD (+) or denotes the treatment were EV (-)

Akt has a known inhibitory effect on GSK-3β activity through phosphorylation at GSK-3β-Ser9. Therefore, we determined if Akt expression or activity might underlie the observed difference in p-Ser9 GSK-3β levels in miR-210 OE transfected AC-16 cardiomyocytes exposed to DOX. Therefore, we quantified the levels of Akt protein in lysates derived from DOX-treated AC-16 cardiomyocytes, either transfected with OE vector or a KD vector. We found no difference in protein expression levels of Akt across the two experiments (Fig. 5A and B). Transfection with a miR-210 OE vector caused, however, a significant increase in p-Ser473 Akt as compared to the EV control group after DOX treatment (*p* < 0.01; Fig. 5C). Meanwhile, transfection with a miR-210 KD vector caused a significant decrease in p-Ser473 as compared to the EV control group after DOX treatment (Fig. 5D). Ser473 Akt phosphorylation status was also confirmed by western blot (Fig. 5E-G). Taken together these data display that OE of miR-210 causes increased activation of Akt whereas miR-210 KD inhibits Akt.

### Predicted miR-210 target genes and their association to Akt

**Fig. 6:**
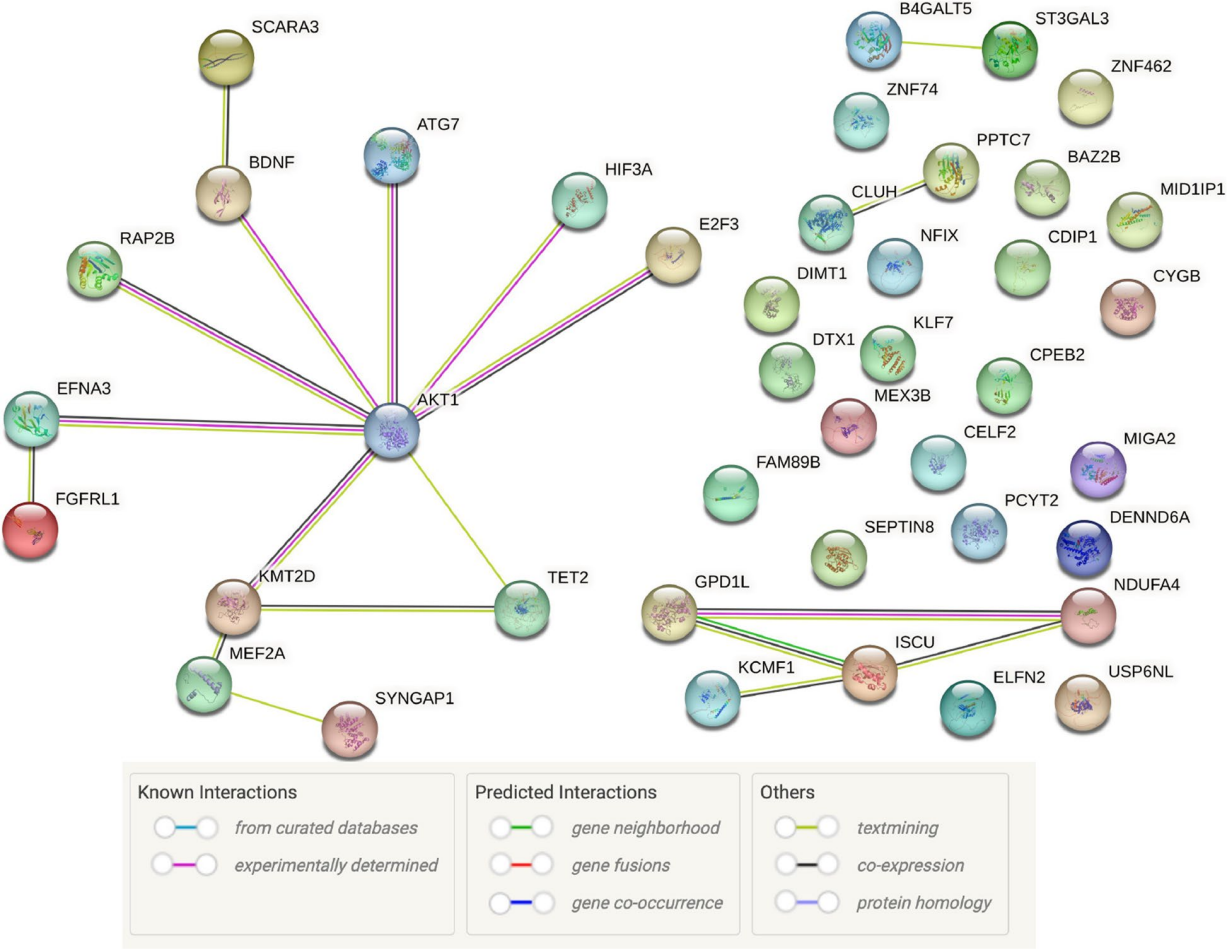
Bioinformatic analysis of predicted miR-210-3p targets in relation to AKT signaling. Predicted gene targets of hsa-miR-210-3p were identified using the TargetScanHuman 8.0 database, which revealed 42 transcripts with conserved binding sites (see Supplementary Table S1). These transcripts were analyzed alongside AKT serine/threonine kinase 1 using the STRING v12.0 database to assess potential protein–protein interactions. The analysis was performed using a medium confidence threshold (interaction score ≥ 0.4) and restricted to evidence-based interaction sources. AKT1 was found within a distinct interaction cluster that included the following proteins with experimentally validated associations: Ephrin-A3 (EFNA3), RAP2B Member Of RAS Oncogene Family (RAP2B), Brain-Derived Neurotrophic Factor (BDNF), Autophagy Related 7 (ATG7), Hypoxia Inducible Factor 3 Subunit Alpha (HIF3A), Lysine Methyltransferase 2D (KMT2D), and E2F Transcription Factor 3 (E2F3), indicated by pink edges. Additional proteins, Scavenger Receptor Class A Member 3 (SCARA3), Tet Methylcytosine Dioxygenase 2 (TET2), Myocyte Enhancer Factor 2 A (MEF2A), Synaptic Ras GTPase Activating Protein 1 Homolog (SYNGAP1), and Fibroblast Growth Factor Receptor Like 1 (FGFRL1), were identified as having indirect associations with AKT1, based on curated databases and text mining (green edges).Remaining predicted targets not associated with AKT1 are displayed on the right-hand side of the network. Edge colors and styles represent the type and confidence of interaction evidence, including known interactions (curated databases, experimental data), predicted interactions (gene neighborhood, gene fusion, co-occurrence), and others (text mining, co-expression, protein homology). Solid lines indicate intra-cluster connections, while dotted lines represent inter-cluster associations. The interaction network was generated using the STRING v12.0 platform (https://string-db.org/)

Our experimental findings indicate that miR-210-3p modulates DOX-induced apoptotic cell death primarily through enhancing AKT phosphorylation at Ser473, rather than altering total AKT protein expression. This suggests that AKT is not a direct target of miR-210-3p, but may be regulated indirectly via upstream effectors. To explore this possibility, we conducted a bioinformatic analysis to identify predicted direct targets of miR-210-3p that may influence AKT signaling. Several predicted target genes were found to have an experimentally determined connection to Akt. These included Ephrin-A3 (EFNA3), RAP2B Member Of RAS Oncogene Family (RAP2B), Brain-Derived Neurotrophic Factor (BDNF), Autophagy Related 7 (ATG7), Hypoxia Inducible Factor 3 Subunit Alpha (HIF3A), Lysine Methyltransferase 2D (KMT2D), and E2F Transcription Factor 3 (E2F3). Others were found to have a more indirect link to Akt (based on textmining; Fig. 6). While the rest were not identified as having a connection with Akt.

## Discussion

Doxorubicin (DOX) is a potent chemotherapeutic agent, but its clinical use is limited by dose-dependent cardiotoxicity [5, 12]. In this study, we demonstrate that miR-210 exerts a protective effect against DOX-induced cardiomyocyte injury by reducing both general and apoptotic cell death in AC-16 cells. Our findings suggest that miR-210 plays a protective role during DOX-induced stress in human AC-16 cardiomyocytes, in line with previous reports examining the role of miR-210 under other stress-induced conditions such as oxygen–glucose deprivation/reperfusion and anoxia [60, 61]. Furthermore, the effects observed by miR-210 modulation in the present study are in line with previous studies demonstrating that miR-210 promotes the survival and proliferation of human cardiomyocytes upon ischemia/reperfusion injury [62], affects the viability of neural progenitor cells in hypoxic conditions where OE miR-210 enhances cell viability, while KD leads to decreased viability under identical hypoxic stress [35].

Additionally, earlier research has indicated that DOX-induced lipid peroxidation, a process linked to ROS production and subsequent cardiotoxicity, may account for the observed increase in LDH release [63]. In this context, numerous studies have demonstrated that the overexpression of miR-210 is associated with decreased ROS levels [64–66]. This reduction in ROS subsequently diminishes the production of malondialdehyde, a lipid peroxidation byproduct that functions as a mitochondrial toxin by impairing respiratory function and inhibiting mitochondrial enzyme activities [67]. Moreover, prior research has elucidated the function of miR-210 in cellular adaptation to inflammatory and stress conditions, underscoring its capacity to attenuate cellular apoptosis [68]. Furthermore, studies have shown that miR-210 enhances cell survival under DOX-induced oxidative stress by reducing apoptosis and increasing cell viability. It modulates genes involved in oxidative stress response and apoptosis, targeting key molecules and pathways to inhibit pro-apoptotic proteins and enhance antioxidant enzyme activity, thereby reducing ROS levels and protecting cells from oxidative damage [64, 65, 69]. miR-210 overexpression has also been shown to reduce cell death in embryonic cardiomyocytes by lowering ROS levels through Akt [66], which align with the findings of the effect on Akt phosphorylation in the present study.

The ROS, DNA double-strand breaks, lipid peroxidation and other molecular events triggered by DOX lead to induction of apoptosis [12, 13, 18, 19, 67]. In neonatal rat cardiomyocytes, miR-210 has been shown to decrease mitochondrial ROS production under antimycin A-treated and hypoxic conditions [66]. Additionally, in an in vitro oxygen glucose deprivation/reperfusion injury model, caspase-3 activity was reduced in cardiomyocytes transfected with miR-210 mimic [60]. Data in the present study show that miR-210 overexpression significantly attenuated, whereas its knockdown exacerbated, caspase-3 activity in DOX-treated cardiomyocytes, indicating that miR-210 modulates apoptotic signaling under DOX-induced stress, consistent with its previously described cytoprotective role.

Increased inhibitory phosphorylation at p-Ser9 of GSK-3β mediates a decrease in the activation of the intrinsic apoptotic pathway [41]. Here we show that miR-210 OE increased p-Ser9 phosphorylation of GSK-3β, suggesting reduced intrinsic apoptosis in DOX-treated AC-16 cardiomyocytes, consistent with GSK-3β being a distal target of miR-210 [37]. of miR-210 elevated LDH release and caspase-3 activity but did not further reduce the already low p-Ser9 levels, indicating additional pathways contribute to apoptosis upon miR-210 KD.

To explore the mechanism, we examined Akt, a known regulator of GSK-3β. Total Akt expression was unaffected by DOX or miR-210 modulation, but p-Ser473 Akt was significantly reduced by DOX, in line with previous findings in heart tissue, rat cardiomyocytes, and H9c2 cells [42]. OE of miR-210 restored p-Ser473 Akt levels, whereas KD further decreased them, supporting a role for miR-210 in regulating Akt activity post-translationally under DOX stress. Although other kinases can phosphorylate GSK-3β at Ser9, the PI3K-Akt pathway is a key regulator [41], and Akt activation via p-Ser473 is critical for cardiomyocyte survival under stress. Thus, miR-210 appears to inhibit apoptosis in DOX-treated AC-16 cells by enhancing p-Ser473 Akt and consequently increasing inhibitory phosphorylation of GSK-3β.

A limitation of our study is that we did not examine the upstream regulatory cascades responsible for the direct targeting of miR-210, which ultimately influences p-Ser473 Akt. Elucidating these pathways is essential to confirm the precise mechanisms by which miR-210 interacts with Akt and its predicted mRNA targets under DOX-induced stress. To address this gap, we performed an in silico analysis to identify potential miR-210 target genes with experimentally validated associations to AKT signaling. This analysis revealed seven promising candidates: KMT2D, EFNA3, E2F3, RAP2B, BDNF, ATG7, and HIF3A. Shangguan et al. [70] demonstrated that knockdown of KMT2D in murine chondrogenic cells increases p-Ser473 AKT levels. Since KMT2D is a predicted target of miR-210, its downregulation may contribute to the observed AKT activation. Similarly, EFNA3 has been implicated in miR-210-mediated protection against oxygen–glucose deprivation/reperfusion-induced apoptosis in neonatal rat and AC-16 cardiomyocytes [62]. Zhuang et al. [71] showed that suppression of EFNA3 enhances PI3K and AKT phosphorylation, supporting its role as a plausible intermediary. In the case of E2F3, Xu et al. [72] found that its overexpression reduces PI3K and AKT phosphorylation and promotes apoptosis in a rat model of coronary heart disease. miR-210 is predicted to target E2F3, and its suppression could alleviate E2F3-mediated inhibition of the PI3K-Akt pathway. Xu et al. [72] demonstrated that E2F3 overexpression reduces phosphorylation of PI3K and Akt compared to controls, indicating pathway inactivation. Given that E2F3 is a predicted miR-210 target and its overexpression suppresses PI3K-Akt signaling, the observed increase in p-Ser473 Akt in our study may result from miR-210-driven downregulation of E2F3.

Cui et al. [73] reported that RAP2B suppression by miR-205 in murine TM3 cells decreased PI3K, AKT, and p-AKT levels. If miR-210 similarly targets RAP2B, it is unlikely to explain the increased AKT phosphorylation observed in our study. A comparable rationale applies to BDNF, as Zhao et al. [74] demonstrated that elevated BDNF expression enhances AKT phosphorylation and promotes cardiomyocyte survival; thus, miR-210-mediated BDNF downregulation would be expected to reduce AKT activity rather than increase it. Finally, ATG7, an autophagy-related enzyme, was shown by Zhao et al. to influence p-Ser473 Akt levels through the Jun-Phosphatase and Tensin Homolog pathway in HepG2 cells, suggesting a potential indirect link between miR-210 and Akt signaling via autophagy regulation. [75]. Silencing ATG7 with small interfering RNA increased p-Ser473 Akt levels, suggesting that ATG7 may negatively regulate Akt phosphorylation. As ATG7 is a predicted miR-210 target, this could explain the elevated p-Ser473 Akt observed when miR-210 was upregulated in DOX-treated AC-16 cardiomyocytes. In contrast, evidence from an in vitro chronic intermittent hypoxia model in rat pulmonary artery smooth muscle cells showed that HIF3A silencing decreased p-PI3K and p-Akt levels [76]. In that study, HIF3A was identified as a miR-485-5p target, and modulation of miR-485-5p altered HIF3A expression accordingly. If miR-210 similarly targets HIF3A in AC-16 cells, its suppression would likely reduce, not increase, Akt phosphorylation—contrary to our findings. Taken together, based on literature and bioinformatic analysis, KMT2D, EFNA3, E2F3, and ATG7 emerge as the most plausible direct miR-210 targets contributing to the observed increase in p-Ser473 Akt in DOX-treated AC-16 cardiomyocytes. Further experimental validation is needed to confirm these interactions and clarify underlying mechanisms.

While the cardioprotective role of miR-210 has previously been demonstrated in models of hypoxia, ischemia/reperfusion, and other stress conditions [30–33, 35–37, 60, 61], the present study is, to our knowledge, the first to establish a mechanistic link between miR-210 and the Akt/GSK-3β signaling axis in the context of DOX-induced cardiotoxicity. This distinction is important, as anthracycline cardiotoxicity involves unique molecular stressors such as DNA damage and mitochondrial dysfunction. From a translational standpoint, miR-210 presents as a promising biomarker for the early detection of chemotherapy-induced cardiotoxicity, owing to its sensitivity to cellular stress and its involvement in key survival pathways. However, therapeutic modulation of miR-210, particularly through the PI3K/Akt axis, requires careful consideration in oncology settings given previous indications of the implication in tumor progression, angiogenesis, and resistance to therapy across multiple cancer types [30, 46, 47]. Therefore, rather than representing a limitation, this dual role highlights the importance of context-specific strategies. Future research should aim to delineate tissue-specific effects and explore delivery methods that maximize cardiac protection while minimizing oncologic risks, thereby advancing the feasibility of miR-210-based interventions in cardio-oncology.

In this study, AC-16 cardiomyocytes were selected as the experimental cell line due to their human genome, ease of genetic modification, and capacity to produce sufficient biological material for in-depth analyses of molecular signaling processes. However, it is important to note that this cell line lacks certain inherent properties of adult human primary cardiomyocytes, such as a fully developed structural and functional electrophysiological machinery. Consequently, the findings presented in this study should be further validated in future research using either primary human cardiomyocytes, neonatal cardiomyocytes or preclinical in vivo models to enhance translational relevance. It is important to note that the DOX concentration used in this study (5 µM) exceeds the peak plasma concentrations typically observed in human cancer patients, which range from 0.5 to 1 µM [77]. While this concentration was selected based on prior dose–response data in AC-16 cardiomyocytes [48], as well as other data using the same concertation in AC-16 cardiomyocytes [78] or higher (10 µM) [79], it represents a supraphysiological exposure. This may amplify cellular stress responses and apoptotic signaling beyond what is observed clinically. Therefore, the results should be interpreted with caution regarding their direct applicability to human physiology. Future studies incorporating lower, clinically relevant DOX concentrations (0.5–1 µM) would be valuable to confirm the cardioprotective effects of miR-210 under conditions that more closely mimic patient exposure. Such pilot experiments could substantially strengthen the translational potential of the findings and help delineate dose-dependent effects of miR-210 modulation.

## Conclusion

This study demonstrates that miR-210 exerts a protective effect on AC-16 cardiomyocytes exposed to DOX for 24 h by reducing overall cell death. miR-210 OE under these conditions decreased caspase-3 activity significantly, indicating reduced apoptotic cell death. This effect was associated with increased levels of p-Ser473 Akt and p-Ser9 GSK-3β. Conversely, miR-210 KD resulted in the opposite, detrimental effect in DOX-stimulated cardiomyocytes. The present findings substantiate a novel role for miR-210 in mitigating DOX-induced cardiomyocyte death, laying the foundation for further studies to explore its potential as a therapeutic target.

## Data Availability

The data and material that support the findings of this study are available from the corresponding author, [MAH], upon reasonable request.
